# The ST131 *Escherichia coli H*22 subclone from human intestinal microbiota: Comparison of genomic and phenotypic traits with those of the globally successful *H*30 subclone

**DOI:** 10.1186/s12866-017-0984-8

**Published:** 2017-03-27

**Authors:** Marie-Hélène Nicolas-Chanoine, Marie Petitjean, Azucena Mora, Noémie Mayer, Jean-Philippe Lavigne, Olivier Boulet, Véronique Leflon-Guibout, Jorge Blanco, Didier Hocquet

**Affiliations:** 1 Service de Microbiologie, Hôpital Beaujon, AP-HP, Clichy, France; 2 Faculté de Médecine Paris Diderot, Paris, France; 3 INSERM UMR 1137, Université Paris 7, Paris, France; 4 Laboratoire d’Hygiène Hospitalière, CHRU Besançon, Besançon, France; 5 UMR 6249 Chrono-Environnement, Besançon, France; 6 Laboratorio de Referencia de E. coli (LREC), Facultad de Veterinaria, Universidad de Santiago de Compostela, Lugo, Spain; 7 Institut National de la Santé et de la Recherche Médicale, U1047, Université de Montpellier, UFR de Médecine, Nîmes, France; 8 Service de Microbiologie, CHU Carémeau, Nîmes, France; 9 Laboratoire d’Analyse Médicale du Centre, Coulommiers, France

**Keywords:** *E. coli* ST131, *H*22 genome, Sugar metabolism, Mannose-binding FimH region, Biofilm, Subclades B, Plasmid replicons

## Abstract

**Background:**

In 2006, we found healthy subjects carrying ST131 *Escherichia coli* in their intestinal microbiota consisting of two populations: a subdominant population of fluoroquinolone-resistant *E. coli* belonging to subclone *H*30 (*H*30-R or subclade C1), the current worldwide dominant ST131 subclone, and a dominant *E. coli* population composed of antibiotic-susceptible *E. coli* belonging to subclone *H*22 (clade B)*,* the precursor of subclone *H*30. We sequenced the whole genome of fecal *H*22 strain S250, compared it to the genomes of ExPEC ST131 *H*30-Rx strain JJ1886 and commensal ST131 *H*41 strain SE15, sought the *H*22-*H*30 genomic differences in our fecal strains and assessed their phenotypic consequences.

**Results:**

We detected 173 genes found in the Virulence Factor Database, of which 148 were shared by the three ST131 genomes, whereas some were genome-specific, notably those allowing determination of virotype (D for S250 and C for JJ1886). We found three sequences of the FimH site involved in adhesion: two in S250 and SE15 close and identical, respectively, to that previously reported to confer strong intestinal adhesion, and one in JJ1886, corresponding to that commonly present in uropathogenic *E. coli.* Among the genes involved in sugar metabolism, one encoding a gluconate kinase lacked in S250 and JJ1886. Although this gene was also absent in both our fecal *H*22 and *H3*0-R strains, *H*22 strains showed a higher capacity to grow in minimal medium with gluconate. Among the genes involved in gluconate metabolism, only the *ghrB* gene differed between S250/*H*22 and JJ1886/*H*30-R strains, resulting in different gluconate reductases. Of the genes involved in biofilm formation, two were absent in the three genomes and one, *fimB*, in the JJ1886 genome. Our fecal *H*30-R strains lacking intact *fimB* displayed delayed biofilm formation relative to our fecal *H*22 strains. The *H*22 strains differed by subclade B type and plasmid content, whereas the *H*30-R strains were identical.

**Conclusions:**

Phenotypic analysis of our fecal strains based on observed genomic differences between S250 and JJ1886 strains suggests the presence of traits related to bacterial commensalism in our *H*22 strains and traits commonly found in uropathogenic *E. coli* in our *H*30-R strains.

**Electronic supplementary material:**

The online version of this article (doi:10.1186/s12866-017-0984-8) contains supplementary material, which is available to authorized users.

## Background

Phylogenetic group B2, sequence type (ST) 131 *Escherichia coli* has been a worldwide dominant human extra-intestinal pathogenic *E. coli* (ExPEC) since the beginning of the 2000s, and is among those resistant to fluoroquinolones and/or producing the extended-spectrum β-lactamase (ESBL) CTX-M-15 [1]. Its dominance was shown to be driven by the expansion of a subclone harboring type 1 fimbriae-encoding *fimH* allele 30 (subclone *H*30), comprised of mostly strains resistant to fluoroquinolones (*H*30-R) [2]. Within this subclone, a subgroup called *H*30-Rx, mostly comprising strains resistant to fluoroquinolones and producing CTX-M-15, has quickly emerged and disseminated [2]. The evolutionary history of clone ST131 revealed that before the emergence and dissemination of subclone *H*30 (also called clade C), the ST131 population consisted of mostly two subclones called *H*22 (clade B) and *H*41 (clade A), with subclone *H*22 comprised of mostly fluoroquinolone-susceptible isolates [3–5]. It also revealed that subclone *H*22 was the precursor of subclone *H*30 and that separate F-type plasmids have shaped the evolution of subclone *H*30 [2, 3, 5, 6]. The most recent ST131 *E. coli* phylogenetic reconstruction carried out by Ben Zakour et al. using 3779 non-recombinant single nucleotide polymorphisms (SNP) found in the high quality genomes of 172 clade B and C strains, identified five B subclades, for which the independent evolutionary trajectories are characterized by successive insertions and a recombination from ancestral *H*22 (subclade B1) [7]. Subclade B2 is characterized by a Flag-2 locus insertion, subclade B3 by a Flag-2 locus insertion and *par-C2* recombination, subclade B4 by Flag-2 locus and GI-PheV insertions, and subclade B5 by Flag-2 locus and Phi3 insertions. Other insertions, notably that of GI-PheV, recombination events, and mutations in *gyrA* and *parC* occurred within strains of subclade B5, resulting in their evolution to clade C and subclades C1 (*H*30-R) and C2 (*H*30-Rx). Epidemiologically, the 172 isolates consisted of mostly those from North America obtained between 1948 and 2011, with most collected between 2000 and 2010, irrespective of the clade and subclade types. Since the first description of the *fimH* lineage in 2013 [3], the *fimH* type has been found in several epidemiological studies published on ExPEC or fecal ST131 isolates [8–17]. However, these have mostly concerned fluoroquinolone-resistant and /or ESBL-producing isolates. In 2006, we retrieved non-ESBL-producing ST131 *E. coli* isolates, susceptible or resistant to fluoroquinolones, from the intestinal microbiota of 7% of healthy subjects living in the Paris area [18]. The fluoroquinolone-resistant isolates accounted for a subdominant *E. coli* population in four independent healthy subjects, whereas those susceptible to fluoroquinolones accounted for the dominant *E. coli* population in three other independent subjects. The latter finding strongly suggests that ST131 *E. coli* is part of the normal intestinal microbiota of humans. In the present study, we aimed to determine whether these isolates belong to different *H* subclones, based on their fluoroquinolone-susceptibility pattern. The dominant fluoroquinolone-susceptible ST131 *E. coli* populations belonged to subclone *H*22. Assembled and annotated genomes of *H*22 strains were not available when we performed the present study. Thus, we first sequenced the whole genome of one of our commensal *H22* strains (S250) and compared it to the genome of two reference ST131 strains: the ExPEC *H*30-Rx strain JJ1886 and the commensal *H*41 strain SE15. This genome comparison focused on virulence factor (VF)-encoding genes and those encoding processes or structures (sugar metabolism, biofilm formation, and the FimH mannose-binding site) known to be involved in the adaptation of the bacteria to different environments including human intestine. The four fluoroquinolone-resistant fecal ST131 isolates were *H*30-R strains. Thus, we compared them to the three fecal *H*22 strains considering the genomic differences identified between *H*22 strain S250 and *H*30-Rx strain JJ1886, and analyzed the phenotypic impact of some of these differences.

## Methods

### Bacterial strains

The seven fecal ST131 isolates (strains 02, 39, 183, 187, 196, 208, and S250) obtained from the intestinal microbiota of seven healthy subjects living in the Paris area in 2006 were included in the study. They display serotype O25:H4, do not produce ESBL, and are either susceptible (196, 208 and S250) or resistant (02, 39, 183 and 187) to fluoroquinolones [18]. The fluoroquinolone-resistant strains were retrieved by plating the feces of healthy subjects on nalidixic acid-containing plates. The fluoroquinolone-susceptible strains accounted for the dominant *E. coli* population of three subjects. We previously assessed the virulence potential of strain S250 in the *Caenorhabditis elegans* and zebrafish models and analyzed its genome by optical mapping. This strain had a level of virulence similar to that of the multidrug resistant ST131 isolates, with which it shared 86% genome similarity [19]. We also included the *E. coli* K-12 MG1655 reference strain in the study as a control in the experiments assessing the use of gluconate as the sole source of carbon.

### *fimH* type

*fimH* typing was based on the internal 489-nucleotide (nt) sequence of the *fimH* gene as previously described [20].

### Antibiotic susceptibility

Antibiotic susceptibility was determined by the agar diffusion method and interpreted following the 2015 EUCAST recommendations (*www.eucast.org*). The following antibiotics were tested: amoxicillin, amoxicillin + clavulanic acid, ciprofloxacin, gentamicin, amikacin, cotrimoxazole and fosfomycin.

### Molecular analysis of resistance mechanisms

The genes encoding resistance to amoxicillin (TEM and SHV enzymes) were identified by PCR and sequencing methods as previously described [21]. The *qnr* genes encoding plasmid-mediated resistance to fluoroquinolones were tested and the quinolone resistance determining region (QRDR) of the genes *gyrA*, *gyrB*, *parC* and *parE* amplified and sequenced using methods previously described [21–23]. The QRDRs of our strains were compared with those of the fluoroquinolone-susceptible reference strain *E. coli* K-12 MG1655 [GenBank: CP014225.1] and the allelic *gyrA* and *parC* profiles were compared with those previously described for ST131 isolates [3].

### Sequencing and analysis of the whole genome of *H*22 strain S250

The complete genomic sequence was determined for *H*22 strain S250. Total DNA was extracted using the Qiagen Blood & Cell Culture DNA Mini Kit (Qiagen, Courtaboeuf, France). Libraries were constructed using Nextera technology and sequenced on an Illumina HiSeq-2000 using a 2 × 100 nucleotides (nt) paired-end strategy. All reads were processed to remove low quality or artefactual nucleotides, using sequentially sickle (www.github.com/najoshi/sickle), AlienTrimmer [24] and fqDuplicate (ftp.pasteur.fr/pub/gensoft/projects/fqtools). Read pairs were assembled using clc_assembler from the CLC Genomics Workbench analysis package (www.clcbio.com/products/clc-genomics-workbench). All contigs of ≥500 nt were reordered and reoriented, using the genomic sequence of strain *E. coli* K-12 MG1655 as a reference, with Mauve Contig Mover [25]. The reordered contigs were analyzed. The genome of *H*22 strain S250 was compared to that of two reference ST131 strains, JJ1886 (*H*30-Rx) [GenBank: CP006784.1] and SE15 (*H*41) [GenBank: AP009378.1], focusing on VF-encoding genes and genes involved in sugar metabolism, biofilm formation, and methylation. We downloaded VFs (www.mgc.ac.cn/VFs/) available from the Virulence Factor Database (VFDB) and searched the three genomes for their presence using Prodigal v2.6.1 [26] and clustered them at 90% identity using CD-hit v4.6 [27]. We extracted the sequences of the genes involved in biofilm formation in strain *E. coli* K12 BW25113 [28] from its genome [GenBank: CP009273.1] and clustered them at 90% identity with CD-hit. We assessed the percentage identity between the genes from *E. coli* K12 BW25113 and the three studied genomes. Using the NBCI basic local alignment search tool (BLAST), we blasted genes from *E. coli* K12 MG1655 against the S250, JJ1886, and SE15 genomes to verify the possible absence of any genes involved in biofilm formation. We then searched the genomes of ST131 strains S250, JJ1886, and SE15 for genes involved in sugar metabolism as defined by Maltby et al. [29] in commensal strains *E. coli* HS [GenBank: CP000802.1] and *E. coli* Nissle 1917 [GenBank: CP007799.1] using BLAST. Moreover, we also searched for all the genes involved in gluconate metabolism (main pathway: *gntR*, *gntT*, *gntU*, *gntP*, *gntK*; secondary pathway: *idnT*, *idnDOTR*; gene *idnK* (*gntV*) which plays a role in the two pathways; the Entner-Doudoroff pathway: *edd* and *eda*; additional genes involved in other pathways: *kdgT*, *kdgK*, *gnd*, *tkrA*, *ghrB*, *kduD*, *dkgA* and *dkgB*) [30], as well as the 100 base pairs (bps) upstream of the start codon of each, in the genome of strains S250 and JJ1886. The nucleotide sequence of the 100 bp-upstream regions and the sequences of the deduced proteins for each gene of the strains S250 and JJ1886 were compared. We also searched for genes encoding methyltransferases in ST131 strain EC958 [GenBank: HG941718.1] [31] in the genome of ST131 strains S250, JJ1886, and SE15 using BLAST.

### Subclade B and clade C typing

According to the work of Ben Zakour et al. [7], we determined the subclade B type of *H*22 strain S250 and the clade C type of strain JJ1886 by direct blasting of the genes of the Flag-2 locus (Flag-2 locus from strain *E. coli* 042 [EMBL: CR 753847]), Phi3 (from strain EC958), and GI-PheV (from strain JJ1886) against the genome of strains S250 and JJI886. We then determined the type of subclade B displayed by strain S250 and the two other fecal *H*22 strains by PCR using primers specific for the Flag-2 locus, Phi3, and GI-PheV (Additional file 1: Table S1) and our *H*30-R fecal strains as a positive control.

### Plasmid content

The plasmid content of *H*22 strain S250 was determined using the PlasmidFinder system [32] and the FII, FIA, and FIB formula of the detected IncF plasmid by PCR-based replicon typing (http://pubmlst.org/plasmid/). The latter method was also applied to the two remaining fecal *H*22 strains and the four fecal *H*30-R strains.

### ExPEC status and virotype

According to the study of Johnson and Adam, ExPEC status is defined by the presence of ≥2 VF genes among the following genes: *pap*, *sfa/focDE*, *afa/draBC*, *iutA*, and *kpsMT II* [33]. As the latter gene is not included in the VFDB, we searched for it by blasting the *kpsMT II* genes [GenBank: X53819] against the S250, JJ1886, and SE15 genomes. Based on the study of Blanco et al., the major virotypes of *E.coli* ST131 are defined using four genes as follows: virotype A = *afa*FM955459^+^ *iroN* ^−^ *ibeA* ^−^ *sat* ^+/−^, virotype B = *afa*FM955459^−^ *iroN* ^+^ *ibeA* ^−^ *sat* ^+/−^, virotype C = *afa*FM955459^−^ *iroN* ^−^ *ibeA* ^−^ *sat* ^+^, and virotype D = *afa*FM955459^+/−^ *iroN* ^*+/*−^ *ibeA* ^+^ *sat* ^*+/*−^ [34]. As *afa*FM955459 is not included in the VFDB, we searched for it by blasting the *afa*FM955459 operon [EMBL: FM955459] against the S250, JJ1886, and SE15 genomes [35]. Then, classic multiplex PCR was used to search for genes encoding ExPEC-associated VFs (Additional file 2: Table S2) [34] in the seven fecal strains to confirm the results provided by the direct genome analysis of strain S250 and to determine the VF profile, virotype, and ExPEC status of the six remaining fecal strains.

### FimH structure

According to the study of Sokurenko et al., the amino acid variations observed within the sequence of the adhesin, FimH, of type 1 fimbriae, result in different levels of binding to mono-mannose (M_1_) structures, whereas they have no impact on the normal high level of binding to tri-mannose (M_3_) structures [36]. By measuring the ratio of M_1_/M_3_ binding in different experimental models, they defined low M_1_-binding (M_1_/M_3_ < 0.1) and high M_1_-binding (M_1_/M_3_ > 0.90) FimH phenotypes and showed that they are related to specific FimH sequences [37]. Therefore, the deduced protein sequence of FimH of *H*22 strain S250 was compared with that of *H*30-Rx ST131 strain JJ1886 [GenBank: AGY86963.1], *H*41 ST131 strain SE15 [GenBank: BAI57801.1], phylogroup A *E. coli* strain MG1655 [GenBank: AMC97175.1], and two other phylogroup B2 strains: ST73 strain CFT073 [GenBank: AAN83822.1] and ST131 uk_P46212 [GenBank: ALT52319.1]. The deduced protein sequence of FimH was then characterized for the six remaining fecal strains by using primers for which the sequences have been previously published [20].

### Amplification and sequencing of the *idnK* (*gntV*) and *ghrB* genes of the seven fecal strains and growth with gluconate as the sole carbon source

The *idnk* (*gntV*) gene, encoding a thermosensitive D-gluconate kinase, and the *ghrB* gene, encoding a gluconate reductase, were amplified with primers indicated in Additional file 1: Table S1. Additional primers were used to sequence the *ghrB* gene (Additional file 1: Table S1). We evaluated the ability of the seven strains to grow in the presence of gluconate as the sole carbon source, as previously described [38]. Experiments were conducted three times independently and all incubations were performed overnight at 37 °C with shaking (150 rpm). Briefly, bacteria were first cultured in Mueller Hinton broth before washing twice in minimal media M63 [15 mM (NH_4_)_2_SO_4_; 100 mM KH_2_PO_4_; 0.002 mM FeSO_4_ (7H_2_O)]. We inoculated 5 ml of M63 supplemented with 0.2% glucose (Sigma-Aldrich, France) with 10 μl of the washed bacteria. One ml of this culture was washed twice in M63. We transferred 10 μl of this washed culture into 5 ml of M63 with 0.2% gluconate (Sigma-Aldrich, Saint-Quentin Fallavier, France). The cultures were then adjusted to 0.002 at OD_600_ in 30 ml fresh M63 with 0.2% gluconate and incubated at 37 °C with shaking (150 rpm). We estimated the bacterial growth after 48 h by measuring the OD_600_ [39]. Tukey’s test was used for intergroup comparisons and R software for statistical analyses. *P* values <0.01 were considered to be statistically significant.

### Amplification and sequencing of the *fimB* gene and kinetics of biofilm formation in the seven fecal strains

The *fimB* gene was amplified and sequenced with the primers indicated in Additional file 1: Table S1. The kinetics of early biofilm formation was assessed using the BioFilm Ring Test® (BioFilm Control, Saint Beauzire, France), as described [40]. Briefly, standardized bacterial cultures were incubated at 37 °C in a 96-well microtiter plate in the presence of magnetic beads. At various time points, the plates were placed onto a magnetic test block and put in the reader. The images of each well before and after magnetic attraction were analyzed using BioFilm Control software that gives a BioFilm Index (BFI). The BFI was converted into the proportion of immobilized beads relative to a reference condition (% RBI) using the formula: % RBI = √[(1-(BFI_assay_-BFI_min_)/(BFI_control_-BFI_min_)] ×100, where BFI_assay_ is the BFI of the tested strain, BFI_control_ is the BFI of the control, corresponding to the maximum BFI, and BFI_min_ is the minimal observed BFI when all the beads are blocked. The more RBI approaches a value of 1, the more the biofilm is fully formed (beads are immobilized). Three experiments were performed in duplicated per strain and per incubation time. The kinetics of biofilm formation were compared using a two-way ANOVA followed by Dunnett’s multiple comparisons test.

## Results

### *fimH* type, antibiotic susceptibility, molecular analysis of resistance mechanisms, and allelic profiles of the *gyrA* and *parC* genes

The three fluoroquinolone-susceptible strains (S250, 208 and 196) were of the *fimH*22 type. All but one were susceptible to all of the antibiotics tested (Table 1). *H*22 strain 196 was resistant to both amoxicillin and cotrimoxazole. The four fluoroquinolone-resistant strains (187, 183, 39 and 02), which were all resistant to amoxicillin but susceptible to the other antibiotics tested, were of the *fimH*30 type (*H*30-R) (Table 1). QRDR nucleotide sequence analysis showed that the three *H*22 strains displayed *gyrA*1a and *parC*1 alleles, whereas the four *H*30-R strains displayed the *gyrA*1AB allele, encoding amino acid substitutions S83 L and D87N, and the *parC*1aAB allele, encoding amino acid substitutions S80I and E84V (Table 1). We found the substitution I529L in ParE in the seven fecal strains (Table 1). None of these strains harbored plasmid-mediated *qnr* genes. *H*22 196 and the four *H*30-R strains that were resistant to amoxicillin harbored a TEM-1-encoding gene (Table 1).

**Table 1:** Characterization of the seven fecal strains of *E. coli* ST131

Strain/ *FimH* type	Susceptibility							β-lactamase	Allele type^a^(amino acid substitution)			Flag-2	Phi3	GI-PheV	Subclade	IncF plasmid replicon
	AMX	AMC	CIP	GEN	AMK	SXT	FOS		*gyrA*	*parC*	*parE*					
S250/*H*22	S	S	S	S	S	S	S	−	1a	1	NA (I529L)	−	−	−	B1	F89:A-:B62
208/*H*22	S	S	S	S	S	S	S	−	1a	1	NA (I529L)	+	−	−	B2	−
196/*H*22	R	S	S	S	S	R	S	TEM-1	1a	1	NA (I529L)	+	−	+	B4	F24:A-:B6
187/*H*30	R	S	R	S	S	S	S	TEM-1	1AB (S83 L/D87N)	1aAB (S80I/E84V)	NA (I529L)	+	+	+	C1	F1:A2:B20
183/*H*30	R	S	R	S	S	S	S	TEM-1	1AB (S83 L/D87N)	1aAB (S80I/E84V)	NA (I529L)	+	+	+	C1	F1:A2:B20
39/*H*30	R	S	R	S	S	S	S	TEM-1	1AB (S83 L/D87N)	1aAB (S80I/E84V)	NA (I529L)	+	+	+	C1	F1:A2:B20
02/*H*30	R	S	R	S	S	S	S	TEM-1	1AB (S83 L/D87N)	1aAB (S80I/E84V)	NA (I529L)	+	+	+	C1	F1:A2:B20

^a^according to reference [3], *AMX* amoxicillin, *AMC* amoxicillin + clavulanic acid, *CIP* ciprofloxacin, *GEN* gentamicin, *AMK* amikacin, *SXT* cotrimoxazole, *FOS* fosfomycin, *R* resistant, *S* susceptible, −: absence, +: presence, *NA* not available

### Genomic and phenotypic characterization

We assembled the whole genome sequence of the fecal *H*22 strain S250 into 50 contigs and analyzed and compared it with those of the ExPEC *H*30-Rx strain JJ1886 and the commensal *H*41 strain SE15.

#### Subclade B and C type

We were unable to find the genes composing the Flag-2 locus in the genome of *H*22 strain S250 using BLAST. We only found a fragment of approximately 1700 bp which was very similar to the end of the first gene, *IfhA,* and another of 769 bp similar to the end to the last gene, *lafU*, of the Flag-2 locus (data not shown). We found neither Phi3 nor GI-PheV in the genome of *H*22strain S250, whereas we found them, as well as the Flag-2 locus, in the genome of JJ11886. The use of specific primers allowed us to confirm the absence of these genetic elements in *H*22 strain 250 and to detect the Flag-2 locus in *H*22 strains 196 and 208, as well as GI-PheV in *H*22 strain 196. These three genetic elements were amplified from our four *H*-30R strains (Table 1).

#### VF-encoding genes

**Fig. 1:**
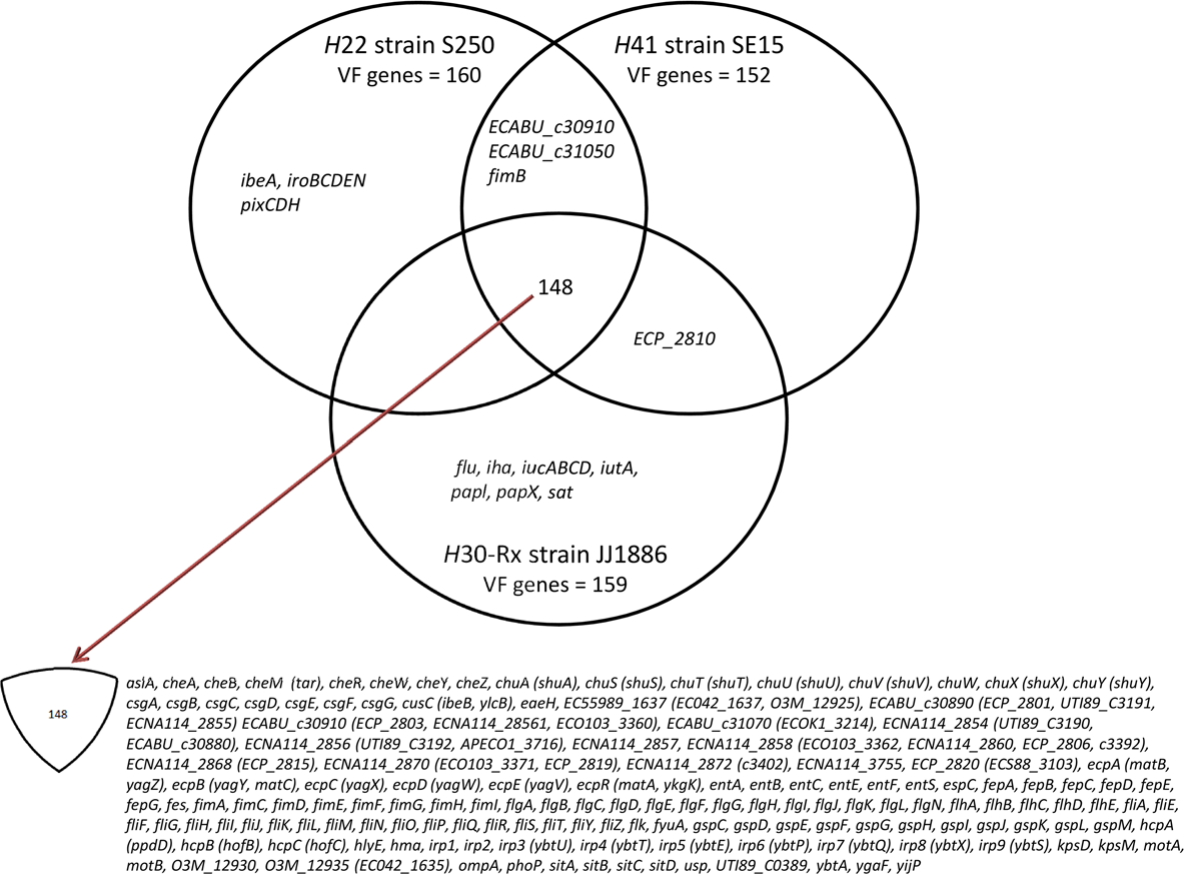
List of genes found in the genomes of *H*22 strain S250, *H*30-Rx strain JJ1186 and, *H*41 strain SE15. Each strain is represented by a circle and each gene commonly found in the genome of two strains or in the genome of the three strains is indicated in the corresponding intersecting regions, whereas genes specific for each strain are indicated in the section of the circle not shared with another circle. The 148 genes present in the three strains are listed at the bottom of the figure

Using a gene identity level of ≥90%, a total of 173 genes among the 2520 *Escherichia* sp. VF-encoding genes of the VFDB was identified in the genomes of the three ST131 strains studied. *H*22 strain S250, *H*30-Rx strain JJ1886, and *H*41 strain SE15 had 160, 159, and 152 VF-encoding genes, respectively. The three ST131 genomes shared 148 virulence genes (Fig. 1). Nine genes were found specifically in *H*22 strain S250: the five *iroBCDEN* genes that encode proteins related to a catecholate siderophore, the three *pixCDH* genes encoding Pix pilus adhesion, and the *ibeA* gene involved in invasion of brain endothelium (Fig. 1). Ten genes were specifically found in *H*30-RX strain JJ1886: the four *iucABCD* and *iutA* genes encoding proteins involved in the binding and transport of iron, the *pap1 and papX* genes encoding *pap* operon regulatory proteins, the *sat* gene encoding a toxin, the *flu* gene encoding autotransporter protein Ag43a, and the *iha* gene encoding the adhesion-siderophore receptor. Three genes, including the *fimB* gene, were present in strains *H*22 S250 and *H*41 SE15, but not *H*30-RX strain JJ1886 (Fig. 1). The ECP_2810 gene, encoding a Val-Gly Repeats-related protein, was present in *H*41 strain SE15 and *H*30-RX strain JJ1886.

#### Virotype and ExPEC status

The VF genes identified in the VFDB would suggest virotype D (*ibeA* ^+^, *iroN* ^*+*^, and *sat* ^−^) for strain S250 and virotype C (*sat* ^+^, *ibeA* ^*−*^, and *iroN* ^−^) for strain JJ1886. These virotypes were confirmed by the absence of the *afaFM955459* operon in the genome of strains S250 and JJ1886, shown using BLAST. We were unable to determine a virotype for strain SE15, as none of the four genes were detected in the genome of this strain. Combining VFDB-based and BLAST analysis of the *kpsMT II* gene, we found that strains S250 and SE15 did not display an ExPEC status, as they harbored only one (*kpsMT II*) of the genes used to define this status. Multiplex PCR classically used to search for genes encoding ExPEC-associated VF (Additional file 2: Table S2) to the seven fecal strains showed that the two remaining fecal *H*22 strains displayed virotype D, as strain S250, and the four fecal *H*30-R strains virotype C, as strain JJ1186 (Table 2). It also showed that only virotype C *H*30-R strain 39 displayed an ExPEC status related to the presence of the *iutA* and *kpsMT II* genes. We found 11 of the amplified VF genes (*fimH, matB, pet, chuA, fyuA, irp2, sitA, traT, malX, usp* and *ompT)* in all but one (strain *H*22 208) strain, five (*F10 papA*, *iha*, *sat*, *iucD* and *iutA*) in only *H*30-R strains, and four (*cdt*, *iroN*, *iss* and *ibeA*) in only *H*22 strains. The number of amplified VF-encoding genes varied from 15 to 17 in *H*30-R strains and 13 to 16 in *H*22 strains.

**Table 2:** Virulence factor-encoding genes, virotype, and ExPEC status of human fecal ST131 *Escherichia coli H22* and *H*30 subclones

Strain	*fimH*	Virulence factor-encoding gene	Virotype	ExPEC status
S250	*H*22	*fimH, matB, pet, iroN, chuA, fyuA, irp2, sitA, kpsMII-K5, iss, traT, ibeA, malX, usp, ompT*	D	-
208	*H*22	*fimH, matB, cdtB, pet, chuA, fyuA, irp2, sitA, kpsMII-K5, ibeA, malX, usp, ompT*	D	-
196	*H*22	*fimH, matB, cdtB, pet, iroN, chuA, fyuA, irp2, sitA, kpsMII-K5, iss, traT, ibeA, malX, usp, ompT*	D	-
187	*H*30	*fimH, F10 papA, iha, matB, sat, pet, iucD, iutA, chuA, fyuA, irp2, sitA, traT, malX, usp, ompT*	C	-
183	*H*30	*fimH, F10 papA, iha, matB, sat, pet, iucD, iutA, chuA, fyuA, irp2, sitA, traT, malX, usp, ompT*	C	-
39	*H*30	*fimH, F10 papA, iha, matB, sat, pet, iucD, iutA, chuA, fyuA, irp2, sitA, kpsMII-K5, traT, malX, usp, ompT*	C	+
02	*H*30	*fimH, F10 papA, matB, sat, pet, iucD, iutA, chuA, fyuA, irp2, sitA, traT, malX, usp, ompT*	C	-

#### Deduced protein FimH

**Fig. 2:**
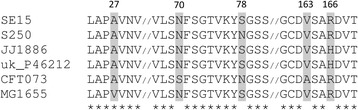
Alignment of FimH sequence from various *Escherichia coli* strains. Phylogroup B2 *E. coli*: ST73 strain CFT073, *H*30 ST131 JJ1886, *H*30 ST131 uk_P46212, *H*22 ST131 S250 and *H*41 ST131 SE15; phylogroup A: MG1655. “1” indicates the start of the FimH protein. Amino acid positions involved in binding to mono-mannose and tri-mannose structures are indicated in *grey*

As indicated in Fig. 2, the deduced protein sequence of adhesin FimH of representatives of *H*30-Rx ST131 *E. coli* (strains JJ1886 and uk_P46212), *H*41 ST131 *E. coli* (strain SE15), *H*22 ST131 *E. coli* (strain S250), UPEC (ST73 strain CFT073), and *E. coli* K12 (strain MG1655) displayed amino acid differences at the positions previously shown to characterize different M_1_/M_3_ ratio phenotypes, contributing to different levels of colonization of different niches [37]. Thus, we identified the sequence -A27, N70 and S78 or N78 and S70, V163, and R166- corresponding to the lower M_1_/M_3_ ratio (0.08-0.09) in the study of Sokurenko et al. in strain SE15. We found the substitutions A27V and V163A, that are each related to a ten-fold increase in the M_1_/M_3_ ratio in the Sokurenko et al., study in strain MG1655 and strain CFT073, respectively. We also found the substitution R166H related to a M_1_/M_3_ ratio of 0.33 in the same study in *H*30-Rx strains JJ1886 and uk_P46212. *H*22 strain S250 displayed a sequence not published in the Sokurenko et al. study as residue N was present at both positions 70 and 78. However, we found none of the substitutions known to induce an increase in the M_1_/M_3_ ratio in the FimH sequence of strain S250. We found the same feature in the two other fecal *H*22 strains, whereas the FimH sequence of our four *H*30-R strains was identical to that of *H*30-Rx strains JJ1886 and uk_ P46212 (data not shown).

#### Genes involved in sugar metabolism and growth with gluconate as the sole carbon source

**Fig. 3:**
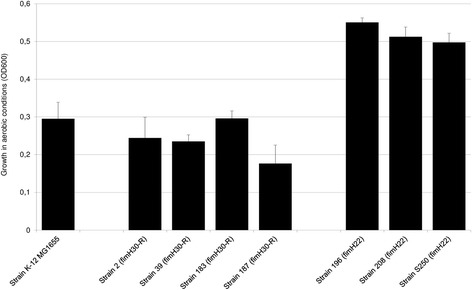
Growth of human fecal subclone *H*30 and subclone *H*22 strains of ST131 *Escherichia coli* in minimal medium plus 0.2% gluconate. Strain growth was assessed by measuring the OD at 600 nm after 48 h of incubation. Bars represent the standard deviation obtained from three independent experiments

All genes involved in sugar metabolism in commensal *E. coli* strains HS and Nissle were detected in the genome of *H*41 strain SE15, whereas one, the D-gluconate kinase-encoding *idnK* (*gntV*) gene, was not detected in either strain *H*30-Rx JJ1886 or *H*22 S250 (Table 3). Its absence in our six remaining strains was revealed by the PCR-sequencing assay. Therefore, we assessed the ability of our strains to grow with gluconate as the sole carbon source in the absence of the *idnK* (*gntV*) gene and the presence of the *gntK* gene, which both transform D-gluconate to 6P-gluconate. After 48 h of growth, the biomass of our *H*30-R strains was significantly lower than that for our *H*22 strains (Tukey’s test *p* < 0.01) (Fig. 3). This unexpected difference led us to compare the genomes of strains *H*22 S250 and *H*30-Rx JJ1886 focusing on all the genes, other than the *idnk* (*gtnV*) gene, involved in gluconate metabolism, as well as the 100 bp upstream of the start codon of the operons or genes. All were present in the genome of strains S250 and JJ1886. The 100 bp upstream of the start codon and the sequence of the deduced proteins showed 100% identity (data not shown) between these two strains, except for the coding region of the *ghrB* gene, which showed a T968C substitution, resulting in amino acid substitution V323A in strain JJ1886. This substitution was confirmed in our four fecal *H*30-R strains, whereas V323 was identified in the two remaining *H*22 fecal strains.

**Table 3:** Distribution of the genes involved in sugar metabolism in commensal *Escherichia coli* strains HS and Nissle [29] in the genomes of representative *H*30, *H*22, and *H*41 subclones of *Escherichia coli* ST131

Commensal strain/gene	Catabolized sugar	*H*30 JJ1886	*H*22 S250	*H*41 SE15
*E. coli* HS				
*araA*	Arabinose	+	+	+
*araB*	Arabinose	+	+	+
*araD*	Arabinose	+	+	+
*fucK*	Fucose	+	+	+
*galK*	Galactose	+	+	+
*gntK*	Gluconate	+	+	+
*idnK*	Gluconate	−	−	+
*lacZ*	Lactose	+	+	+
*manA*	Mannose	+	+	+
*nagE*	N-acetylglucosamine	+	+	+
*nanA*	N-acetylneuraminate	+	+	+
*nanT1*	N-acetylneuraminate	+	+	+
*rbsK*	Ribose	+	+	+
*uxaC*	Glucuronate	+	+	+
*E. coli* Nissle 1917				
*agaA*	N-acetylgalactosamine	+	+	+
*agaE*	N-acetylgalactosamine	+	+	+
*agaF*	N-acetylgalactosamine	+	+	+
*agaW*	N-acetylgalactosamine	+	+	+

#### Biofilm genotype and phenotype

**Fig. 4:**
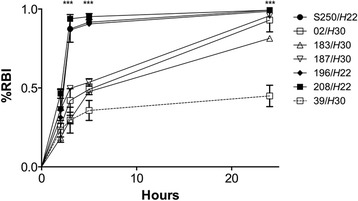
Early biofilm formation by human fecal subclone *H*30 and subclone *H*22 strains of ST131 *Escherichia coli*. BioFilm Control® image analysis software was used and results are expressed as the proportion of immobilized beads relative to reference conditions (% RBI) according to incubation time. Bars represent the standard deviation obtained from three independent experiments performed in duplicate. The biofilm is more fully formed (beads are immobilized) as RBI approaches a value of 1. Significant differences between *H*22 and *H*30 using Dunnett’s test are indicated by * (*p* < 0.05), ** (*p* < 0.01) and *** (*p* < 0.001)

The expression of a wide panel of genes can influence biofilm formation [28]. We specifically searched for these genes in the genome of strains JJ1886, S250, and SE15 (Additional file 3: Table S3). Two genes of the biofilm gene panel, *fliC* and *fliD*, were missing from the three tested genomes. We aligned the *fliC* and *fliD* genes from *E. coli* strain MG1655 onto the three genomes (JJ1886, S250 and SE15) to verify their absence and found deletions within *fliC* and the absence of *fliD* in the three strains. An intact version of the *fimB* gene was present in strains *H*22 S250 and *H*41 SE15, but it was disrupted in *H*30-Rx JJ1886 due to the insertion of *IS*3-like, as previously described in various *H*30 strains [34, 41]. The difference in the structure of the *fimB* gene in strains *H*30-Rx JJ1886 and *H*22 250 led us to sequence the *fimB* gene of the remaining fecal strains. We found that the *fimB* nucleotide sequence in the four *H*30-R strains was identical to that of *H*30-Rx strain JJ1886, whereas that of the two remaining *H*22 strains was identical to that of strain S250. Niba et al. previously reported that the deletion of *fliC* and/or *fliD* induces a substantial decrease in biofilm formation, whereas deletion of *fimB* results in in its near complete absence (Additional file 3: Table S3). We thus tested whether this *fimB* polymorphism affects biofilm formation. Kinetic measurements of early biofilm formation showed that the three *H*22 strains formed biofilms significantly earlier (*p* < 0.0001) than the four *H*30-R strains (Fig. 4). Of note, *H*30-R strain 39, which was the single fecal strain with an ExPEC status, showed significantly (*p*< 0.0001) slower biofilm production (incomplete biofilm formation after 24 h) than the other *H*30-R strains.

#### Genes encoding methyltransferases

We found all but one of the ten methyltransferases, previously identified in the genome of *H*30 ST131strain EC958 [31], in the genome of *H*30-Rx strain JJ1886 (Table 4). We found six in the genome of *H*41 strain SE15 and only three in the genome of *H*22 strain S250 (Table 4).

**Table 4:** Distribution of the 10 methyltransferases previously described in *H*30 strain EC958, [31] in the genomes of representative *H*30, *H*22, and *H*41 subclones of *Escherichia coli* ST131

Methyltransferase (*H*30 EC958)	*H*30 JJ1886	*H*22 S250	*H*41 SE15
M.EcoMIV	+	−	−
M.EcoMV	+	−	−
M.EcoMDcm	+	+	+
EcoMIII	+	−	+
M.EcoMVI	+	+	+
M.EcoMDam	+	+	+
M.EcoMVII	−	−	−
M1.EcoMI	+	−	+
M2.EcoMI	+	−	+
M.EcoMII	+	−	−
Total number	9	3	6

+: presence,-: absence

#### Plasmid content

We detected an IncFII plasmid in contig 4 and an IncFIB element in contig 41 of the S250 genome using PlasmidFinder. Using NCBI BLAST and the plasmid MSLT typing system, we identified the replicon alleles F89 and B62 in the whole genome of strain S250. We confirmed this result for strain S250 by PCR-sequencing, and also obtained the following results for the six remaining fecal strains: F1:A2:B20 for the four *H*30-R strains, no IncF plasmid for *H*22 strain 208, and F24:B6 for *H*22 strain 196 (Table 1).

## Discussion

CTX-M-15 producing, fluoroquinolone-resistant ST131 *E. coli*, which has been shown to be a worldwide human ExPEC since the beginning of the 2000s, has also been shown to colonize the human digestive tract [1]. In 2006, we found healthy subjects with a subdominant intestinal population of fluoroquinolone-resistant ST131 *E. coli*, as well as those with a dominant intestinal population of ST131 *E. coli* susceptible to fluoroquinolones [18]. The latter finding suggests that ST131 *E. coli* may be a human intestinal commensal. Here, we first showed that our three antibiotic-susceptible fecal ST131 *E. coli* strains displayed *fimH*22, whereas the four resistant to fluoroquinolones displayed *fimH*30 (*H*30-R). We thus centered our study on the comparison between these two groups of strains focusing on processes and structures involved in environmental adaptation, considering that subclone *H*22 is the precursor of subclone *H*30, which emerged at the end of 1990s [3, 7]. We first sequenced the whole genome of one of our fecal *H*22 strains, strain S250, as there were no assembled and annotated genomes of *H*22 strains when we started our work. In contrast, there were sequenced whole genomes of representative multidrug resistant *H*30 ExPECs, such as *H*30-RX strain JJ1886 and a commensal ST131 *E. coli*, strain SE15, belonging to another ST131 subclone, characterized by the allele *fimH*41. Comparison of the genomes of *H*22 strain S250, *H*30-Rx strain JJ1886, and *H*41 strain SE15 showed high similarity in terms of the number and types of VF genes from the VFDB. This may explain the similar level of virulence that we found previously between *H*22 strain S250 and an *H*30-Rx strain in the *C. elegans* model [19, 42]. Nevertheless, strains S250 and JJ1886 displayed two different virotypes: D for strain S250 and C for strain JJ1886. Moreover, strain S250 did not display the VF genes required for ExPEC status, whereas strain JJ1886 did [43]. There was also high similarity between the genes involved in biofilm formation in the three genomes [28], except for the *fimB* gene, which was disrupted in the *H*30-Rx strain JJ1886 and intact in both *H*22 strain S250 and *H*41 strain SE15. As the intact *fimB* gene was also absent from our fecal *H*30-R strains and present in our remaining fecal *H*22 strains, we analyzed the kinetics of early biofilm formation in our seven fecal strains. Biofilm formation was significantly delayed in the *H*30-R strains. This is the first study to compare the formation of biofilms by *H*22 and non-ESBL-producing *H*30-R strains [4, 42, 44–46]. Further studies are required to verify the involvement of the *fimB* gene in the two different biofilm phenotypes. Comparison of the genes of the three genomes involved in the metabolism of various sugars showed the absence of one of the two genes encoding gluconate kinases in strains S250 and JJ1886. Although this gene was absent in both our fecal *H*22 and *H3*0-*R* strains, we found that *H*22 strains had a higher capacity to grow in medium with gluconate as the sole carbon source. Thus, gluconate consumption may confer an advantage to *H*22 strains for residing in the human intestine, as gluconate is a component of intestinal mucus [47]. Indeed, it has been shown that *E. coli* laboratory mutants with impaired growth on gluconate are less able to colonize the large intestine of mice [38]. Comparison of all genes, and their promoter regions, involved in the different pathways of gluconate metabolism showed one difference between strains S250 and JJ1886, namely a mutation leading to an amino acid substitution in the 2-keto-D-gluconate reductase GhrB in strain JJ1886. Although this genetic difference was found between the fecal *H*22 and *H*30-R strains, further studies are required to clarify the role of this mutation for the difference in the growth of the two strains when gluconate is the sole carbon source. Another difference we identified between strain S250 and strain JJ1886 concerned the sequence of the type 1 fimbriae FimH adhesin, shown by Sokurenko et al. to be involved in variations of the M_1_/M_3_ ratio associated with tissue tropism and the shift of bacterial adaptation from a commensal to pathological habitat [36, 37]. The FimH sequence of strain JJ1886, which was also present in our *H*30-R fecal strains, corresponded to that identified by Sokurenko et al. in strains with a M_1_/M_3_ ratio = 0.33. This sequence and M_1_/M_3_ ratio are commonly observed in uropathogenic strains [36, 37]. The FimH sequence identified in strain S250 and the other fecal *H*22 strains was not found in the strains studied by Sokurenko et al. and was only found at a low frequency in those studied by Chen et al. [48]. This sequence showed none of the amino acid substitutions involved in the increase of the M_1_/M_3_ ratio. Thus it is likely to be associated with the lowest M_1_/M_3_ ratio shown by Sokurenko et al., which was associated with strong adhesion to intestinal cells [36, 37]. Altogether, although the *H*22 and *H*30-R strains investigated in this work were all isolated from the digestive tract of healthy subjects, only *H*22 strains displayed properties previously shown to be associated with human intestinal commensalism, including competition for nutrients in the intestine (gluconate use) and a high intestinal adhesiveness [49].

We sought to confirm the genetic differences identified here between the lineages *H*22 and *H*30 with the whole genome sequences of ST131 isolates recently deposited on NCBI databases (i.e. 28 *H*22 strains, 9 *H*30 strains, see Additional file 4: Table S4). As expected, we retrieved all but one of these genetic differences in this wider *H*22 and *H*30 strain collection. Indeed, the gluconate reductase-encoding *ghrB* gene in two *H*22 clinical isolates (strain JJ1897 and strain GN02448 from the bioproject PRJNA290784: Additional file 4: Table S4) displayed the same sequence as in all *H*30 strains and not that found in the other *H*22 strains. Of note, these two strains possessed Phi3 and the *par-C*1a variant, the two evolutionary markers present in the *H*22 “intermediate” strain group B0/C0 described by Ben Zakour et al. [7]. The intermediate status was based on the SNP pattern of *H*22 strains which progressively acquired clade C-defining point mutations.

The last comparison made between the S250, JJ1886 and SE15 genomes concerned the genes encoding the 10 methyltransferases previously identified in the genome of *H*30-Rx ST131strain EC958 [31]. We found nine of these genes in strain JJ1886, six in strain SE15, and only three in strain S250. Forde et al. showed that six of the 10 genes detected in strain EC958 are components of defined mobile genetic elements (MGE) that we showed to be absent from the genome of *H*22 strain S250. Indeed, we showed that this strain belongs to subclade B1, meaning tothe ancestor of subclone *H*22 from which evolved the four other B subclades and subclone *H*30 with its subclades C1 and C2. Subclone *H30* arose by acquiring, among others, MGEs near which the methyltransferase-encoding genes are located in *H*30-Rx strain EC958. The three genes identified in strain S250 encode orphan methyltransferases known to be involved in regulation and not methyltransferases involved in restriction modification systems known to inhibit the uptake of non-self DNA and restrict horizontal gene transfer.

We also showed that strain S250 had a *gyrA*1 allele. This allele was not found in the subclade B1 strains studied by Ben Zakour et al., whereas it was found in some of their strains belonging to subclades B4 and B5 [7]. Concerning our two other fecal *H*22 strains that harbored the g*yrA*1a allele, we showed that one belonged to subclade B2 and the other to subclade B4. Plasmid content also varied within our *H*22 strains: no IncF plasmid in one strain and IncF plasmids with different replicons, F24:B6 and F89:B62, in the two remaining *H*22 strains, with allele B62 identified here (strain S250) for the first time. Johnson et al. also found plasmids with FII and FIB replicons in most of their *H*22 strains. However, the two FII and FIB allelic patterns identified by Johnson et al. were different from ours [6]. In contrast, our *H*30-R strains were all of the same subclade with the same plasmid content: subclade C1 and the IncF plasmid with the replicon F1:A2:B20-. This replicon was previously identified in France in CTX-M-27-producing, fluoroquinolone-resistant *H*30 strains isolated from stool of children attending different day-care centers in 2012 [14]. It was also very recently shown by Johnson et al. to be the dominant IncF plasmid replicon type among the fluoroquinolone-resistant *H*30 strains (called *H*30R1) from different sources whereas replicon F2:A1 was shown to be dominant in those *H*30 strains producing CTX-M-15 (*H*30-Rx) [6].

## Conclusions

This study shows, for the first time, that the O25b:H4 ST131 *E. coli* isolates that were carried in the intestinal microbiota of healthy subjects living in the Paris area in 2006 consisted of strains belonging to both the ancient dominant subclone *H*22 and the current dominant subclone *H*30. The genotypic and phenotypic differences identified in this study between these two subclones affect their ability to adapt to the environment. The fecal *H*22 strains displayed traits increasing their ability to colonize human gut, whereas the fecal *H*30-R strains displayed traits allowing extra-intestinal adaptation. Thus, resistance to antibiotics may not be the sole advantage of subclone *H*30-R over its precursor subclone *H*22 in causing worldwide infections.

## Additional files

Additional file 1: Table S1. List of primers designed and used in this study (DOCX 24 kb)

Additional file 2: Table S2. *Escherichia coli* virulence factor encoding genes tested by the PCR method. (DOCX 25 kb)

Additional file 3: Table S3. Distribution and identity percentage of the genes previously shown to be involved in biofilm formation [28] in representatives of different ST131 *H* subclones. (DOCX 41 kb)

Additional file 4: Table S4. *H*22 and *H*30 strains with a whole genome sequence available in NCBI databases either under a circular DNA form or a fragmented DNA form. (DOCX 16 kb)

## Abbreviations

BFI: Biofilm index
BLAST: Basic Local Alignment Search Tool
bp: Base pair
ESBL: Extended-spectrum β-lactamase
ExPEC: Extra-intestinal pathogenic *E. coli*
M_1_: Mono-mannose
M_3_: Tri-mannose
MGE: Mobile genetic elements
NCBI: National Center for Biotechnology Information
nt: nucleotide
QRDR: Quinolone-resistance determining region
SNP: Single-nucleotide polymorphism
ST: Sequence type
VF: Virulence factor
VFDB: Virulence factor database
WGS: Whole genome shotgun

## References

[1] Nicolas-Chanoine MH, Bertrand X, Madec JY (2014). *Escherichia coli* ST131, an intriguing clonal group. Clin Microbiol Rev.

[2] Price LB, Johnson JR, Aziz M, Clabots C, Johnston B, Tchesnokova V, Nordstrom L, Billig M, Chattopadhyay S (2013). The epidemic of extended-spectrum-β-lactamase-producing *Escherichia coli* ST131 is driven by a single highly pathogenic subclone, *H*30-Rx. MBio.

[3] Johnson JR, Tchesnokova V, Johnston B, Clabots C, Roberts PL, Billig M, Riddell K, Rogers P, Qin X (2013). Abrupt emergence of a single dominant multidrug-resistant strain of *Escherichia coli*. J Infect Dis.

[4] Olesen B, Frimodt-Moller J, Leihof RF, Struve C, Johnston B, Hansen DS, Scheutz F, Krogfelt KA, Kuskowski MA (2014). Temporal trends in antimicrobial resistance and virulence-associated traits within the *Escherichia coli* sequence type 131 clonal group and its *H*30 and *H*30-Rx subclones, 1968 to 2012. Antimicrob Agents Chemother.

[5] Stoesser N, Sheppard AE, Pankhurst L, De Maio N, Moore CE, Sebra R, Turner P, Anson LW, Kasarskis A (2016). Evolutionary history of the gobal emergence of the *Escherichia coli* epidemic clone ST131. MBio.

[6] Johnson TJ, Danzeisen JL, Youmans B, Case K, Llop K, Munoz-Aguayo J, Flores-Figueroa C, Aziz M, Stoesser N (2016). Separate F-type plasmids have shaped the evolution of the *H*30 suclone of *Escherichia coli* sequence type 131. mSphere.

[7] Ben Zakour NL, Alsheikh-Hussain AS, Ashcroft MM, Khanh Nhu NT, Roberts LW, Stanton-Cook M, Schembri MA, Beatson SA (2016). Sequential acquisition of virulence and fluoroquinolone resistance has shaped the evolution of *Escherichia coli* ST131. MBio.

[8] Can F, Kurt-Azap O, Ispir P, Nurtop E, Seref C, Loclar I, Aktas ON, Orhan YC, Ergonul O (2016). The clinical impact of ST131 *H*30-Rx subclone in urinary tract infections due to multidrug-resistant *Escherichia coli*. J Glob Antimicrob Resist.

[9] Rodrigues C, Machado E, Fernandes S, Peixe L, Novais A (2016). An update on faecal carriage of ESBL-producing *Enterobacteriaceae* by Portuguese healthy humans: detection of the *H*30 subclone of B2-ST131 *Escherichia coli* producing CTX-M-27. J Antimicrob Chemother.

[10] Ranjan A, Shaik S, Hussain A, Nandanwar N, Semmler T, Jadhav S, Wieler LH, Ahmed N (2015). Genomic and functional portrait of a highly virulent, CTX-M-15-producing *H*30-Rx subclone of *Escherichia coli* sequence type 131. Antimicrob Agents Chemother.

[11] Johnson JR, Johnston B, Kuskowski MA, Sokurenko EV, Tchesnokova V (2015). Intensity and mechanisms of fluoroquinolone resistance within the *H*30 and *H*30Rx subclones of *Escherichia coli* sequence type 131 compared with other fluoroquinolone-resistant *E. coli*. Antimicrob Agents Chemother.

[12] Dahbi G, Mora A, Mamani R, Lopez C, Alonso MP, Marzoa J, Blanco M, Herrera A, Viso S (2014). Molecular epidemiology and virulence of *Escherichia coli* O16:H5-ST131: comparison with *H*30 and *H*30-Rx subclones of O25b:H4-ST131. Int J Med Microbiol.

[13] Rogers BA, Ingram PR, Runnegar N, Pitman MC, Freeman JT, Athan E, Havers S, Sidjabat HE, Gunning E (2015). Sequence type 131 *fimH*30 and *fimH*41 subclones amongst *Escherichia coli* isolates in Australia and New Zealand. Int J Antimicrob Agents.

[14] Blanc V, Leflon-Guibout V, Blanco J, Haenni M, Madec JY, Rafignon G, Bruno P, Mora A, Lopez C (2014). Prevalence of day-care centre children (France) with faecal CTX-M-producing *Escherichia coli* comprising O25b:H4 and O16:H5 ST131 strains. J Antimicrob Chemother.

[15] Zhong YM, Liu WE, Liang XH, Li YM, Jian ZJ, Hawkey PM (2015). Emergence and spread of O16-ST131 and O25b-ST131 clones among faecal CTX-M-producing *Escherichia coli* in healthy individuals in Hunan Province. China J Antimicrob Chemother.

[16] Gurnee EA, Ndao IM, Johnson JR, Johnston BD, Gonzalez MD, Burnham CA, Hall-Moore CM, McGhee JE, Mellmann A (2015). Gut colonization of healthy children and their mothers with pathogenic ciprofloxacin-resistant *Escherichia coli*. J Infect Dis.

[17] Mohamed M, Clabots C, Porter SB, Thuras P, Johnson JR (2016). Isolation and characterization of *Escherichia coli* sequence type 131 (ST131) and other antimicrobial-resistant gram-negative bacilli from clinical stool samples from veterans. Antimicrob Agents Chemother.

[18] Leflon-Guibout V, Blanco J, Amaqdouf K, Mora A, Guize L, Nicolas-Chanoine MH (2008). Absence of CTX-M enzymes but high prevalence of clones, including clone ST131, among fecal *Escherichia coli* isolates from healthy subjects living in the area of Paris. France J Clin Microbiol.

[19] Lavigne JP, Vergunst AC, Goret L, Sotto A, Combescure C, Blanco J, O'Callaghan D, Nicolas-Chanoine MH (2012). Virulence potential and genomic mapping of the worldwide clone *Escherichia coli* ST131. PLoS One.

[20] Weissman SJ, Johnson JR, Tchesnokova V, Billig M, Dykhuizen D, Riddell K, Rogers P, Qin X, Butler-Wu S (2012). High-resolution two-locus clonal typing of extraintestinal pathogenic *Escherichia coli*. Appl Environ Microbiol.

[21] Leflon-Guibout V, Jurand C, Bonacorsi S, Espinasse F, Guelfi MC, Duportail F, Heym B, Bingen E, Nicolas-Chanoine MH (2004). Emergence and spread of three clonally related virulent isolates of CTX-M-15-producing *Escherichia coli* with variable resistance to aminoglycosides and tetracycline in a French geriatric hospital. Antimicrob Agents Chemother.

[22] Guillard T, de Champs C, Moret H, Bertrand X, Scheftel JM, Cambau E (2012). High-resolution melting analysis for rapid characterization of *qnr* alleles in clinical isolates and detection of two novel alleles, *qnr*B25 and *qnr*B42. J Antimicrob Chemother.

[23] Paltansing S, Kraakman ME, Ras JM, Wessels E, Bernards AT (2013). Characterization of fluoroquinolone and cephalosporin resistance mechanisms in *Enterobacteriaceae* isolated in a Dutch teaching hospital reveals the presence of an *Escherichia coli* ST131 clone with a specific mutation in *parE*. J Antimicrob Chemother.

[24] Criscuolo A, Brisse S (2013). AlienTrimmer: a tool to quickly and accurately trim off multiple short contaminant sequences from high-throughput sequencing reads. Genomics.

[25] Rissman AI, Mau B, Biehl BS, Darling AE, Glasner JD, Perna NT (2009). Reordering contigs of draft genomes using the mauve aligner. Bioinformatics.

[26] Hyatt D, Chen GL, Locascio PF, Land ML, Larimer FW, Hauser LJ (2010). Prodigal: prokaryotic gene recognition and translation initiation site identification. BMC Bioinf.

[27] Li W, Jaroszewski L, Godzik A (2001). Clustering of highly homologous sequences to reduce the size of large protein databases. Bioinformatics.

[28] Niba ET, Naka Y, Nagase M, Mori H, Kitakawa M (2007). A genome-wide approach to identify the genes involved in biofilm formation in *E. coli*. DNA Res.

[29] Maltby R, Leatham-Jensen MP, Gibson T, Cohen PS, Conway T (2013). Nutritional basis for colonization resistance by human commensal *Escherichia coli* strains HS and Nissle 1917 against *E. coli* O157:H7 in the mouse intestine. PLoS One.

[30] Gomez KM, Rodriguez A, Rodriguez Y, Ramirez AH, Isturiz T (2011). The subsidiary GntII system for gluconate metabolism in *Escherichia coli:* alternative induction of the *gntV* gene. Biol Res.

[31] Forde BM, Phan MD, Gawthorne JA, Ashcroft MM, Stanton-Cook M, Sarkar S, Peters KM, Chan KG, Chong TM (2015). Lineage-specific methyltransferases define the methylome of the globally disseminated *Escherichia coli* ST131 clone. MBio.

[32] Carattoli A, Zankari E, Garcia-Fernandez A, Voldby Larsen M, Lund O, Villa L, Moller Aarestrup F, Hasman H (2014). *In silico* detection and typing of plasmids using PlasmidFinder and plasmid multilocus sequence typing. Antimicrob Agents Chemother.

[33] Johnson JR, Stell AL (2000). Extended virulence genotypes of *Escherichia coli* strains from patients with urosepsis in relation to phylogeny and host compromise. J Infect Dis.

[34] Blanco J, Mora A, Mamani R, Lopez C, Blanco M, Dahbi G, Herrera A, Marzoa J, Fernandez V (2013). Four main virotypes among extended-spectrum-β-lactamase-producing isolates of *Escherichia coli* O25b:H4-B2-ST131: bacterial, epidemiological, and clinical characteristics. J Clin Microbiol.

[35] Blanco M, Alonso MP, Nicolas-Chanoine MH, Dahbi G, Mora A, Blanco JE, Lopez C, Cortes P, Llagostera M (2009). Molecular epidemiology of *Escherichia coli* producing extended-spectrum β-lactamases in Lugo (Spain): dissemination of clone O25b:H4-ST131 producing CTX-M-15. J Antimicrob Chemother.

[36] Sokurenko EV, Courtney HS, Maslow J, Siitonen A, Hasty DL (1995). Quantitative differences in adhesiveness of type 1 fimbriated *Escherichia coli* due to structural differences in *fimH* genes. J Bacteriol.

[37] Sokurenko EV, Chesnokova V, Dykhuizen DE, Ofek I, Wu XR, Krogfelt KA, Struve C, Schembri MA, Hasty DL (1998). Pathogenic adaptation of *Escherichia coli* by natural variation of the FimH adhesin. Proc Natl Acad Sci U S A.

[38] Leatham MP, Stevenson SJ, Gauger EJ, Krogfelt KA, Lins JJ, Haddock TL, Autieri SM, Conway T, Cohen PS (2005). Mouse intestine selects nonmotile *flhDC* mutants of *Escherichia coli* MG1655 with increased colonizing ability and better utilization of carbon sources. Infect Immun.

[39] D'Argenio DA, Wu M, Hoffman LR, Kulasekara HD, Deziel E, Smith EE, Nguyen H, Ernst RK, Larson Freeman TJ (2007). Growth phenotypes of *Pseudomonas aeruginosa lasR* mutants adapted to the airways of cystic fibrosis patients. Mol Microbiol.

[40] Chavant P, Gaillard-Martinie B, Talon R, Hebraud M, Bernardi T (2007). A new device for rapid evaluation of biofilm formation potential by bacteria. J Microbiol Methods.

[41] Totsika M, Beatson SA, Sarkar S, Phan MD, Petty NK, Bachmann N, Szubert M, Sidjabat HE, Paterson DL (2011). Insights into a multidrug resistant *Escherichia coli* pathogen of the globally disseminated ST131 lineage: genome analysis and virulence mechanisms. PLoS One.

[42] Pantel A, Dunyach-Remy C, Ngba Essebe C, Mesureur J, Sotto A, Pages JM, Nicolas-Chanoine MH, Lavigne JP (2016). Modulation of membrane influx and efflux in *Escherichia coli* sequence type 131 has an impact on bacterial motility, biofilm formation, and virulence in a *Caenorhabditis elegans* model. Antimicrob Agents Chemother.

[43] Owens RCJ, Johnson JR, Stogsdill P, Yarmus L, Lolans K, Quinn J (2011). Community transmission in the United States of a CTX-M-15-producing sequence type ST131 *Escherichia coli* strain resulting in death. J Clin Microbiol.

[44] Hussain A, Ranjan A, Nandanwar N, Babbar A, Jadhav S, Ahmed N (2014). Genotypic and phenotypic profiles of *Escherichia coli* isolates belonging to clinical sequence type 131 (ST131), clinical non-ST131, and fecal non-ST131 lineages from India. Antimicrob Agents Chemother.

[45] Morgand M, Vimont S, Bleibtreu A, Boyd A, Thien HV, Zahar JR, Denamur E, Arlet G (2014). Extended-spectrum β-lactamase-producing *Escherichia coli* infections in children: are community-acquired strains different from nosocomial strains?. Int J Med Microbiol.

[46] Sarkar S, Vagenas D, Schembri MA, Totsika M. Biofilm formation by multidrug resistant *Escherichia coli* ST131 is dependent on type 1 fimbriae and assay conditions. Pathog Dis. 2016;74(3):ftw013 10.1093/femspd/ftw013 26940589

[47] Peekhaus N, Conway T (1998). What's for dinner?: Entner-Doudoroff metabolism in *Escherichia coli*. J Bacteriol.

[48] Chen SL, Hung CS, Pinkner JS, Walker JN, Cusumano CK, Li Z, Bouckaert J, Gordon JI, Hultgren SJ (2009). Positive selection identifies an in vivo role for FimH during urinary tract infection in addition to mannose binding. Proc Natl Acad Sci U S A.

[49] Conway T, Cohen PS (2015). Commensal and pathogenic *Escherichia coli* metabolism in the gut. Microbiol Spectr.

