# Supplementary material for: The ST131 Escherichia coli H22 subclone from human intestinal microbiota: Comparison of genomic and phenotypic traits with those of the globally successful H30 subclone
Source: BMC Microbiol. 2017 Mar 27;17:71. doi: 10.1186/s12866-017-0984-8 (PMC5369007; doi:10.1186/s12866-017-0984-8)
Supplement: Supplementary file 1 — List of primers designed and used in this study (DOCX 24 kb) [file 12866_2017_984_MOESM1_ESM.docx]

**Additional file Table S1.** List of primers designed and used in this study

| Targeted region | Primer | Sequence (5’-3’) |
| --- | --- | --- |
| **Inserted elements** |  |  |
| Phi3 | phi3-F | AATACCCAAATCGCAAGCAC |
|  | phi3-R | ACGACGGAAAATAAGACCGA |
| *lfhB* (Flag-2 locus) | lfhB-F | TCAGCAAAATCAGTCCGTTA |
|  | lfhB-R | GTTTCATTTGCAGGACGTAG |
| *lafA* (Flag-2 locus) | lafA-F | AACACCAATAACGCCTCAAT |
|  | lafA-R | TGTTCAGTTGGCTTGAAATG |
| GI-pheV | GIpheV-F | GCCAGTAATAAACAATGGGC |
|  | GIpheV-R | TTCCTTGGTTGTGTCATACC |
| **Gluconate metabolism** |  |  |
| *idnK* | idnK-F | TATCGCAGCAGGTAAGATGA |
|  | idnK-R | GGTGTTGAAAGCCGATTTTTG |
| *ghrB* | ghrB-F | TCACCTGGCATTCTTCAGTT |
|  | ghrB-R | TTATCGGGCTTTACTCCTCG |
| additional primers for sequencing | ghrB-sqF | CGTTCACCATAAAACACTGG |
|  | ghrB-sqR | AGAGGATGGGCATGTTGAAG |
| ***fimB*** | fimB-F | AGCATGGCGTTTGTATGG |
|  | fimB-R | CCCTGGTATCTCAACTATCTCT |
