# Supplementary material for: The ST131 Escherichia coli H22 subclone from human intestinal microbiota: Comparison of genomic and phenotypic traits with those of the globally successful H30 subclone
Source: BMC Microbiol. 2017 Mar 27;17:71. doi: 10.1186/s12866-017-0984-8 (PMC5369007; doi:10.1186/s12866-017-0984-8)
Supplement: Supplementary file 2 — Escherichia coli virulence factor encoding genes tested by the PCR method. (DOCX 25 kb) [file 12866_2017_984_MOESM2_ESM.docx]

**Additional file 2: Table S2. *Escherichia coli* virulence factor encoding genes tested by the PCR method**

| **Virulence gene** | **Virulence factor** |
| --- | --- |
| **Adhesin** |  |
| *fimH* | Type 1 fimbriae |
| *fimAv_MT78_** | FimA variant MT78 of type 1 fimbriae |
| *F10 papA** | F type |
| *papA* | P fimbriae operon. Major structural subunit. |
| *papC* | P fimbriae operon. Pilus assembly. |
| *papEF* | P fimbriae operon. Minor tip pilins. Connect PapG to PapA. |
| *sfa/focDE* | Central region of *sfa* and *foc* operons |
| *sfaS* | S fimbriae |
| *focG* | F1C fimbriae |
| *afa/draBC* | Dr antigen-specific adhesin operons (AFA, Dr, F1845) |
| *afaFM955459** | Operon *afa* specific for clonal group O25b-ST131 |
| *iha* | Adhesin-siderophore receptor |
| *bmaE** | Blood group M-specific adhesin |
| *gafD** | N-acetyl-D-glucosamine-specific (G, F17c) fimbriae adhesin |
| *hra** | Heat-resistant agglutinin |
| *matB* | Meningitis and temperature-dependent fimbriae |
| **Toxin and serine protease** |  |
| *cnf1* | Cytotoxic necrotizing factor type 1 |
| *cdtB* | Cytolethal distending toxin |
| *sat* | Secreted autotransporter toxin |
| *hlyA* | α-hemolysin |
| *hlyE* | Hemolysin operon |
| *pet* | Plasmid-encoded toxin |
| *vat* | Vacuolating autotransporter toxin. Serine protease |
| *pic* | Protein involved in intestinal colonization. Serine protease |
| *tsh* | Tsh (temperature-sensitive hemagglutinin). Serine protease |
| **Siderophore** |  |
| *iucD* | Ferric aerobactin receptor (iron uptake: transport) |
| *iutA* | Ferric aerobactin receptor (iron uptake: transport) |
| *iroN* | Novel catecholate siderophore receptor. Salmochelin receptor |
| *chuA* | Home binding protein. Outer membrane iron receptor. |
| *fyuA* | Yersiniabactin receptor. Ferric yersinia uptake. |
| *irp2* | Iron-repressible protein |
| *sitA* | Iron–uptake system |
| *ireA* | Siderophore receptor. Iron-responsive element. |
| *eitA** | Iron–uptake system |
| **Capsule** |  |
| *kpsM II** | Group II capsule |
| *kpsM II-K2** | K2 group II capsule |
| *kpsM II-K5** | K5 group II capsule |
| *neuC-K1** | K1 group II capsule |
| *kpsM III** | Group III capsule |
| **Miscellaneous** |  |
| *cvaC** | ColV; on plasmids with traT, iss, and antibiotic resistance |
| *iss** | Increased serum survival (outer membrane protein) |
| *traT** | Surface exclusion, serum survival (outer membrane protein) |
| *ibeA* | IbeA: Invasion of brain endothelium |
| *malX* (PAI)* | Pathogenicity-associated island marker |
| *usp* | Uropathogenic-specific protein (bacteriocin) |
| *ompT** | Outer membrane protease T |

*: gene not included in the Virulence Factor DataBase
