# Supplementary material for: The ST131 Escherichia coli H22 subclone from human intestinal microbiota: Comparison of genomic and phenotypic traits with those of the globally successful H30 subclone
Source: BMC Microbiol. 2017 Mar 27;17:71. doi: 10.1186/s12866-017-0984-8 (PMC5369007; doi:10.1186/s12866-017-0984-8)
Supplement: Supplementary file 3 — Distribution and identity percentage of the genes previously shown to be involved in biofilm formation [28] in representatives of different ST131 H subclones. (DOCX 41 kb) [file 12866_2017_984_MOESM3_ESM.docx]

**Additional file 3: Table S3**. Distribution and identity percentage of the genes previously shown to be

involved in biofilm formation (Niba et al., 2007) in representatives of different ST131 subclones *H*

| Function | Gene | Biofilm (%) when the gene was defective in *E. coli* K12 BW25113 | | Percentage of identity with the gene of strain BW25113 | | |
| --- | --- | --- | --- | --- | --- | --- |
|  |  |  |  | *H*30 JJ1886 | *H*22 S250 | *H*41 SE15 |
|  |  | av | SD |  |  |  |
| Motility |  |  |  |  |  |  |
|  | *flgA* | 16.1 | 7.6 | 97.72 | 97.72 | 97.72 |
|  | *flgB* | 17.5 | 16.4 | 99.28 | 99.28 | 99.28 |
|  | *flgC* | 16.9 | 6.0 | 100 | 100 | 100 |
|  | *flgD* | 15.4 | 10.3 | 99.57 | 99.57 | 99.57 |
|  | *flgE* | 14.0 | 6.5 | 94.03 | 94.03 | 95.00 |
|  | *flgF* | 12.8 | 5.7 | 98.80 | 98.80 | 98.80 |
|  | *flgG* | 20.6 | 16.4 | 100 | 100 | 100 |
|  | *flgH* | 16.8 | 5.4 | 99.57 | 99.57 | 99.57 |
|  | *flgI* | 20.2 | 11.9 | 100 | 100 | 100 |
|  | *flgJ* | 22.5 | 8.1 | 99.36 | 99.36 | 99.36 |
|  | *flgK* | 16.4 | 5.8 | 99.45 | 99.45 | 99.45 |
|  | *flgL* | 10.2 | 4.4 | 98.42 | 98.42 | 98.42 |
|  | *flgN* | 47.1 | 16.3 | 97.10 | 97.10 | 97.10 |
|  | *flhA* | 22.1 | 5.6 | 99.57 | 99.57 | 99.57 |
|  | *flhB* | 16.4 | 5.0 | 99.21 | 99.21 | 99.21 |
|  | *flhC* | 17.7 | 3.5 | 100 | 100 | 100 |
|  | *flhD* | 28.7 | 4.4 | 100 | 100 | 100 |
|  | *flhE* | 56.1 | 27.7 | 94.62 | 94.62 | 93.00 |
|  | *fliA* | 18.9 | 6.9 | 99.58 | 99.58 | 99.58 |
|  | *fliC* | 18.1 | 5.8 | - | - | - |
|  | *fliD* | 13.4 | 5.8 | - | - | - |
|  | *fliE* | 13.8 | 5.7 | 100 | 100 | 100 |
|  | *fliF* | 19.1 | 8.1 | 98.73 | 98.73 | 99.09 |
|  | *fliG* | 14.4 | 2.3 | 100 | 100 | 100 |
|  | *fliH* | 13.6 | 8.7 | 97.81 | 97.81 | 96.93 |
|  | *fliI* | 18.7 | 3.2 | 99.78 | 99.78 | 100 |
|  | *fliJ* | 10.9 | 4.4 | 99.32 | 99.32 | 99.32 |
|  | *fliK* | 19.1 | 4.1 | 98.13 | 98.13 | 97.87 |
|  | *fliL* | 46.6 | 4.4 | 100 | 100 | 100 |
|  | *fliM* | 21.9 | 9.4 | 99.70 | 99.70 | 99.70 |
|  | *fliN* | 18.7 | 4.9 | 96.35 | 96.35 | 96.35 |
|  | *fliO* | 9.5 | 3.8 | 99.17 | 99.17 | 99.17 |
|  | *fliP* | 18.1 | 6.0 | 99.59 | 99.59 | 99.59 |
|  | *fliQ* | 17.0 | 3.7 | 100 | 100 | 100 |
|  | *fliR* | 15.3 | 5.2 | 98.08 | 98.08 | 98.08 |
|  | *fliS* | 26.8 | 6.0 | 99.26 | 99.26 | 99.26 |
|  | *fliT* | 59.0 | 4.9 | 97.52 | 97.52 | 98.35 |
|  | *motA* | 52.5 | 14.5 | 99.32 | 99.32 | 99.32 |
|  | *motB* | 15.9 | 4.4 | 99.03 | 99.03 | 99.03 |
| Type 1 Fimbriae |  |  |  |  |  |  |
|  | *fimA* | 3.9 | 3.9 | 94.51 | 90.66 | 93.00 |
|  | *fimB* | 1.2 | 1.4 | - | 99.50 | 100 |

**Supplemental table 3** Distribution and identity percentage of the genes previously shown to be

involved in biofilm formation (Niba et al., 2007) in representatives of different ST131 subclones *H* (continued)

| Function | Gene | Biofilm (%) when the gene is defective in strain *E. coli* K12 BW25113 (ref) | | Percentage of identity with the gene of strain BW25113 | | |
| --- | --- | --- | --- | --- | --- | --- |
|  |  |  |  | *H*30 JJ1886 | *H*22 S250 | *H*41 SE15 |
|  |  | av | SD |  |  |  |
|  | *fimC* | 1.0 | 1.3 | 99.59 | 99.50 | 99.59 |
|  | *fimD* | 1.4 | 1.9 | 99.32 | 99.17 | 98.86 |
|  | *fimF* | 1.2 | 1.8 | 98.30 | 98.63 | 98.86 |
|  | *fimG* | 17.5 | 15.1 | 98.20 | 98.30 | 98.80 |
|  | *fimH* | 1.1 | 1.5 | 99.33 | 99 | 99.33 |
| Curli |  |  |  |  |  |  |
|  | *csgA* | 32.9 | 4.8 | 97.37 | 97.37 | 97.37 |
|  | *csgB* | 33.1 | 3.3 | 100 | 100 | 100 |
|  | *csgD* | 52.9 | 1.6 | 99.07 | 99.07 | 99.07 |
|  | *csgE* | 43.8 | 7.6 | 100 | 100 | 100 |
|  | *csgF* | 45.2 | 8.4 | 99.28 | 99.28 | 99.28 |
|  | *csgG* | 42.9 | 2.5 | 99.64 | 99.64 | 99.64 |
| LPS |  |  |  |  |  |  |
|  | *lpcA* | 9.6 | 1.0 | 100 | 100 | 100 |
|  | *gmhB* | 24.7 | 4.1 | 98.42 | 98.42 | 98.42 |
|  | *rfaD* | 12.5 | 2.0 | 99.68 | 99.68 | 99.68 |
|  | *rfaE* | 11.4 | 2.3 | 99.79 | 99.79 | 99.79 |
|  | *rfaF* | 13.2 | 2.2 | 99.71 | 99.71 | 99.71 |
|  | *rfaG* | 19.1 | 9.9 | 90.64 | 90.64 | 90.64 |
|  | *rfaH* | 22.7 | 13.7 | 99.38 | 99.38 | 99.38 |
|  | *rfaP* | 12.3 | 6.4 |  |  |  |
| Other |  |  |  |  |  |  |
|  | *btuB* | 72.5 | 27.2 | 98.05 | 98.05 | 98.05 |
|  | *cheZ* | 65.4 | 15.4 | 99.07 | 99.07 | 100 |
|  | *crp* | 8.9 | 4.5 | 100 | 100 | 100 |
|  | *crr* | 72.5 | 10.6 | 100 | 100 | 100 |
|  | *cyaA* | 4.5 | 4.3 | 99.65 | 99.65 | 99.65 |
|  | *degP* | 38.4 | 7.4 | 99.37 | 99.37 | 99.16 |
|  | *dgkA* | 76.7 | 6.3 | 99.18 | 99.18 | 99.18 |
|  | *dnaK* | 46.2 | 5.8 | 100 | 100 | 100 |
|  | *dsbA* | 8.5 | 3.3 | 99.52 | 99.52 | 100 |
|  | *dsbB* | 43.6 | 11.5 | 98.86 | 98.86 | 98.86 |
|  | *galU* | 24.3 | 11.7 | 100 | 100 | 100 |
|  | *gcvA* | 74.6 | 16.9 | 99.67 | 99.67 | 100 |
|  | *greA* | 49.8 | 3.5 | 100 | 100 | 100 |
|  | *hfq* | 43.6 | 4.1 | 100 | 100 | 100 |
|  | *hscB* | 68.0 | 14.6 | 100 | 100 | 100 |
|  | *hsrA/yieO* | 70.8 | 3.7 | 99.58 | 99.58 | 99.37 |
|  | *ihfB* | 2.2 | 2.0 | 100 | 100 | 100 |
|  | *Ion* | 37.3 | 4.3 | 100 | 100 | 100 |
|  | *mdoH* | 60.1 | 10.4 | 99.88 | 99.88 | 99.88 |
|  | *mlrA* | 61.5 | 4.9 | 98.77 | 98.77 | 98.77 |

**Supplemental table 3**. Distribution and identity percentage of the genes previously shown to be involved in biofilm formation (Niba et al., 2007) in representatives of different ST131 subclones *H* (continued)

| Function | Gene | Biofilm (%) when the gene is defective in strain *E. coli* K12 BW25113 (ref) | | Percentage of identity with the gene of strain BW25113 | | |
| --- | --- | --- | --- | --- | --- | --- |
|  |  |  |  | *H*30 JJ1886 | *H*22 S250 | *H*41 SE15 |
|  |  | av | SD |  |  |  |
|  |  |  |  |  |  |  |
|  | *mltE* | 72.0 | 16.6 | 99.01 | 99.01 | 99.01 |
|  | *mog* | 68.3 | 12.6 | 99.49 | 99.49 | 99.49 |
|  | *nagA* | 64.1 | 5.1 | 99.74 | 99.74 | 99.74 |
|  | *yihA/nanC* | 43.5 | 3.8 | 100 | 99.58 | 100 |
|  | *nifU* | 68.6 | 4.1 | 100 | 100 | 100 |
|  | *nlpD* | 60.0 | 4.2 | 99.74 | 99.74 | 99.47 |
|  | *nlpI* | 56.8 | 4.6 | 100 | 100 | 100 |
|  | *ompR* | 47.8 | 3.7 | 100 | 100 | 100 |
|  | *pgi* | 59.2 | 4.0 | 98.00 | 99.82 | 99.82 |
|  | *proQ* | 66.6 | 16.8 | 99.14 | 99.14 | 99.14 |
|  | *ptsI* | 63.4 | 9.3 | 99.65 | 99.65 | 99.65 |
|  | *rcsC* | 72.9 | 3.9 | 99.47 | 99.47 | 99.47 |
|  | *rpmE* | 59.8 | 10.4 | 100 | 100 | 100 |
|  | *rpoS* | 58.6 | 8.6 | 99.64 | 99.70 | 99.70 |
|  | *sdhC* | 73.3 | 5.6 | 99.22 | 99.22 | 99.22 |
|  | *surA* | 3.3 | 2.9 | 100 | 100 | 100 |
|  | *tolA* | 58.6 | 4.1 | 96.33 | 96.33 | 96.33 |
|  | *tolB* | 45.6 | 4.8 | 100 | 100 | 100 |
|  | *tolR* | 45.9 | 2.3 | 100 | 100 | 100 |
|  | *yfgL* | 37.5 | 10.5 | 98.72 | 98.72 | 98.72 |
| Uncharacterized |  |  |  |  |  |  |
|  | *ycfM* | 50.4 | 7.3 | 99.53 | 99.53 | 99.53 |
|  | *yciB/ispZ* | 48.0 | 12.2 | 99.44 | 99.44 | 99.44 |
|  | *yciM* | 39.9 | 14.0 | 99.49 | 99.74 | 99.74 |
|  | *ydaM* | 58.2 | 4.3 | 98.78 | 98.78 | 98.78 |
|  | *yfgA* | 63.3 | 15.1 | 94.07 | 94.07 | 94.07 |
|  | *yhcB* | 68.3 | 19.4 | 100 | 100 | 100 |
|  | *yicO* | 75.7 | 39.0 | 98.87 | 98.87 | 98.87 |
|  | *ynjC* | 41.0 | 7.1 | 96.48 | 96.67 | 96.67 |

av: average; SD: Standard Deviation
