# Supplementary material for: The ST131 Escherichia coli H22 subclone from human intestinal microbiota: Comparison of genomic and phenotypic traits with those of the globally successful H30 subclone
Source: BMC Microbiol. 2017 Mar 27;17:71. doi: 10.1186/s12866-017-0984-8 (PMC5369007; doi:10.1186/s12866-017-0984-8)
Supplement: Supplementary file 4 — H22 and H30 strains with a whole genome sequence available in NCBI databases either under a circular DNA form or a fragmented DNA form. (DOCX 16 kb) [file 12866_2017_984_MOESM4_ESM.docx]

**Additional file 4: Table S4.** *H*22 and *H*30 strains with a whole genome sequence available in NCBI

databases under either a circular DNA form or a fragmented DNA form

| Strain (GenBank accession number) or Bioproject (number  of strains) | Source | Circular DNA | fragmented DNA (contigs) |
| --- | --- | --- | --- |
| *H*22 |  |  |  |
| Strains |  |  |  |
| ZH063 (CP014522.1) | Human clinical isolate | + |  |
| SaT040 (CP014495.1) | Human clinical isolate | + |  |
| G749 (CP014488.1) | Human clinical isolate | + |  |
| JJ1897 (CP013837.1) | Human clinical isolate | + |  |
| Bioprojects |  |  |  |
| PRJNA335932 (2) | Human commensal |  | + |
| PRJNA326050 (1) | Animal |  | + |
| PRJNA322739 (1) | Animal |  | + |
| PRJEB9970 (1) | Human clinical isolate |  | + |
| PRJNA319144 (1) | Animal |  | + |
| PRJNA290784 (4) | Human clinical isolate |  | + |
| PRJNA295914 (14) | Human clinical isolate |  | + |
| *H*30 |  |  |  |
| Strains |  |  |  |
| NCTC13441 (LT632320.1) | Human clinical isolate | + |  |
| Ecol732 (CP015138.1) | Human clinical isolate | + |  |
| JJ1887 (CP014316.1) | Human clinical isolate | + |  |
| ZH193 (CP014497.1) | Human clinical isolate | + |  |
| J52434 (CP013835.1) | Human clinical isolate | + |  |
| CD306 (CP013831.1) | Human clinical isolate | + |  |
| UK-P46212 (CP013658.1) | Human clinical isolate | + |  |
| MNCRE44 (CP010876.1) | Human clinical isolate | + |  |
| EC958 (HG941718.1) | Human clinical isolate | + |  |
